# Supplementary material for: TrEMOLO: accurate transposable element allele frequency estimation using long-read sequencing data combining assembly and mapping-based approaches
Source: Genome Biol. 2023 Apr 3;24:63. doi: 10.1186/s13059-023-02911-2 (PMC10069131; doi:10.1186/s13059-023-02911-2)
Supplement: Supplementary file 1 — Additional file 1: Fig. S1. Schematicrepresentation of TrEMOLO method for TE detection. Fig. S2. Schematic representation to get benchmarking datasets. Fig. S3. Impact of down-sampling on lowfrequency TE detection. Table S1. Primer sequences used for TE detection in Figure 3. Table S2. Primer and probe sequences used for digital PCRexperiment (resumed in Fig. 2). TableS3. Impact of sequencing depth on the detection of OUTSIDER TE insertions. [file 13059_2023_2911_MOESM1_ESM.pdf]

Figure S1

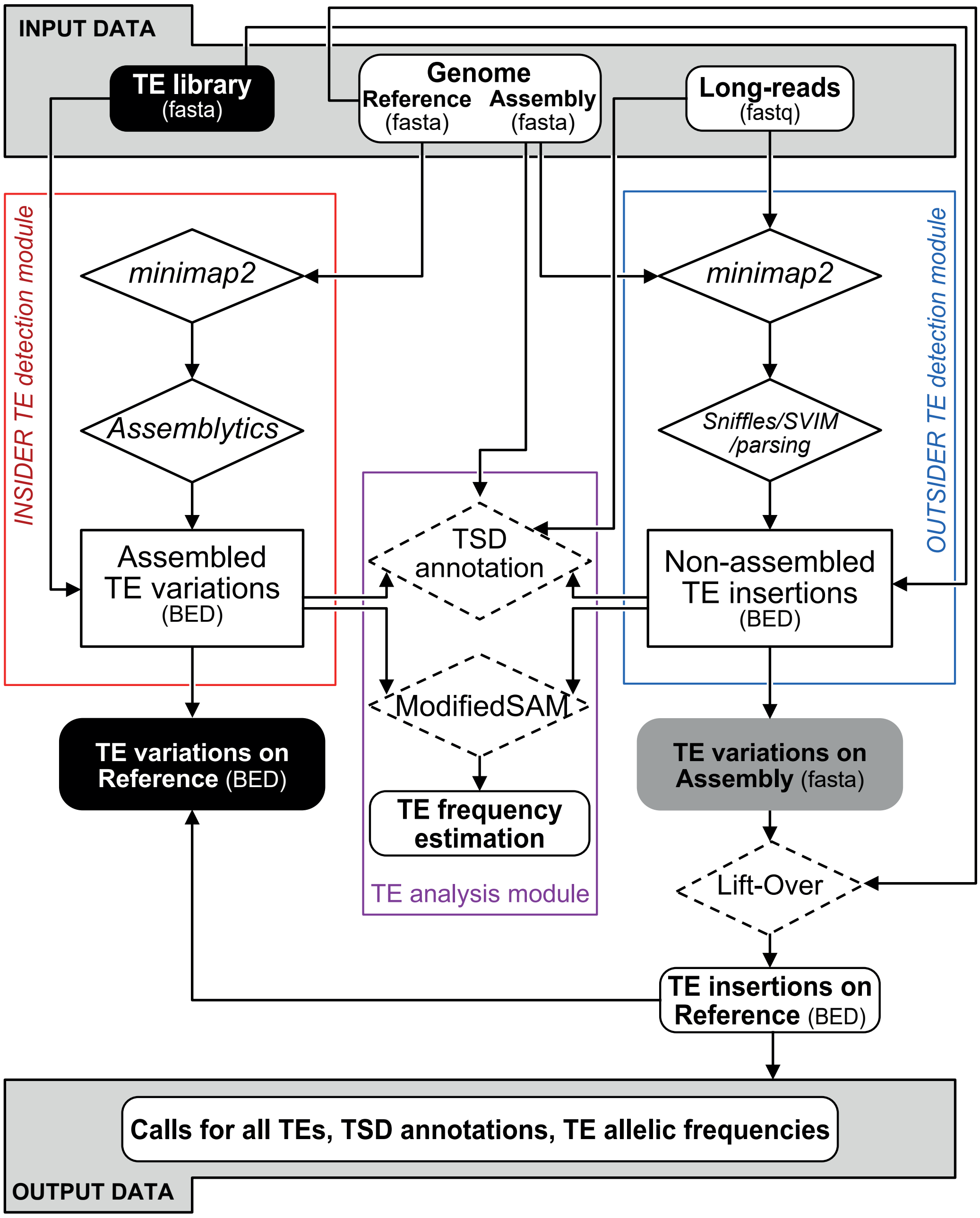

Schematic representation of TrEMOLO method for TE detection

The INSIDER TE module (red) detects TE insertions/deletions in genome assemblies and the OUTSIDER TE module detects TE insertions/deletions by mapping reads on these assemblies (blue). The TE analysis module allows the characterization of the TE insertions by determining the TSDs and estimating the TE frequencies. The coordinates of the insertions can be determined on the reference genome by lift-over. The final output files are calls for all TEs, TSD annotations and TE allelic frequencies.

Figure S2

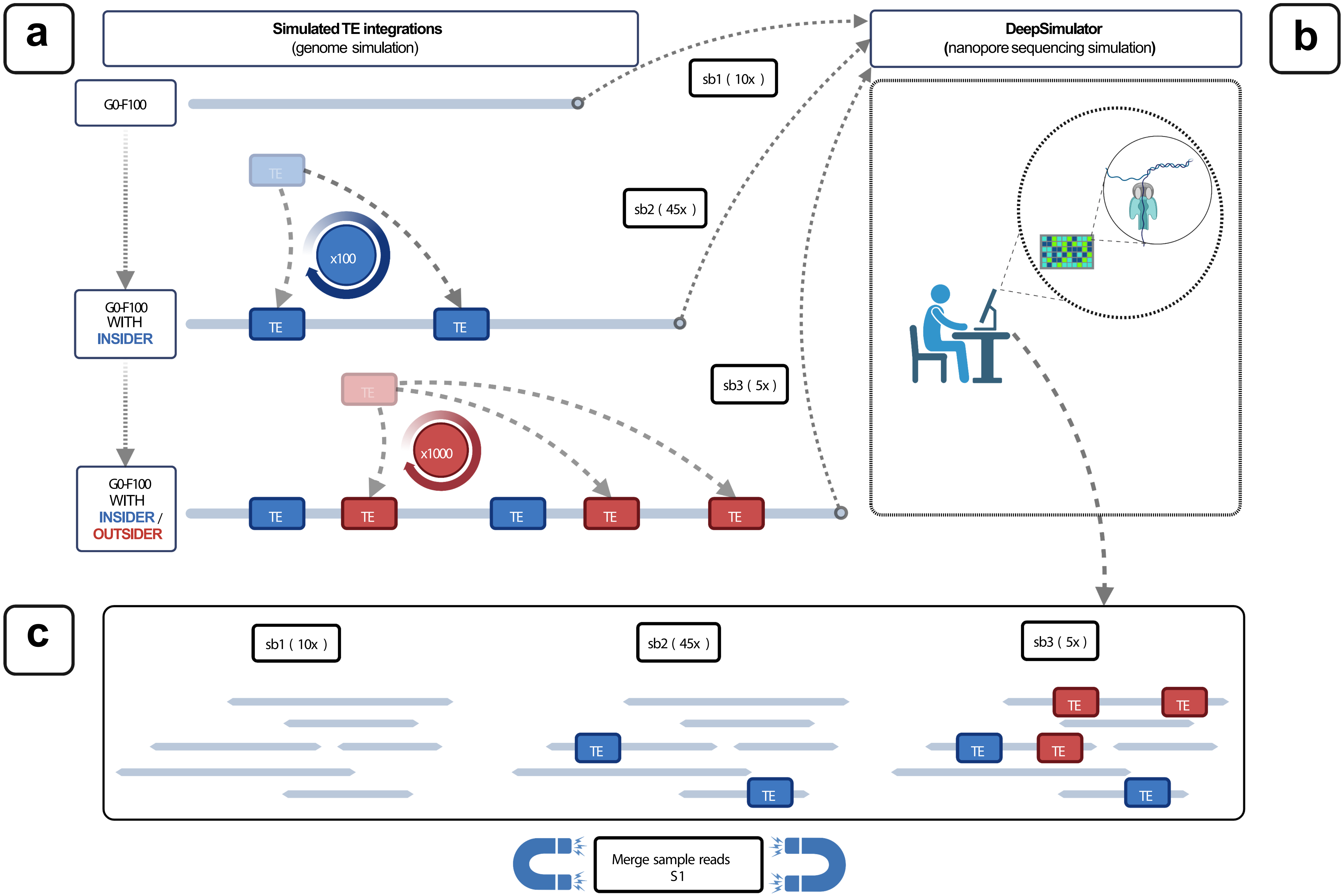

Schematic representation to get benchmarking datasets

a) Genome simulation using the G0-F100 unmasked assembled genome. b) read simulation using DeepSimulator. c) Proportion of the different subsamples in the S1 simulated reads.

Figure S3

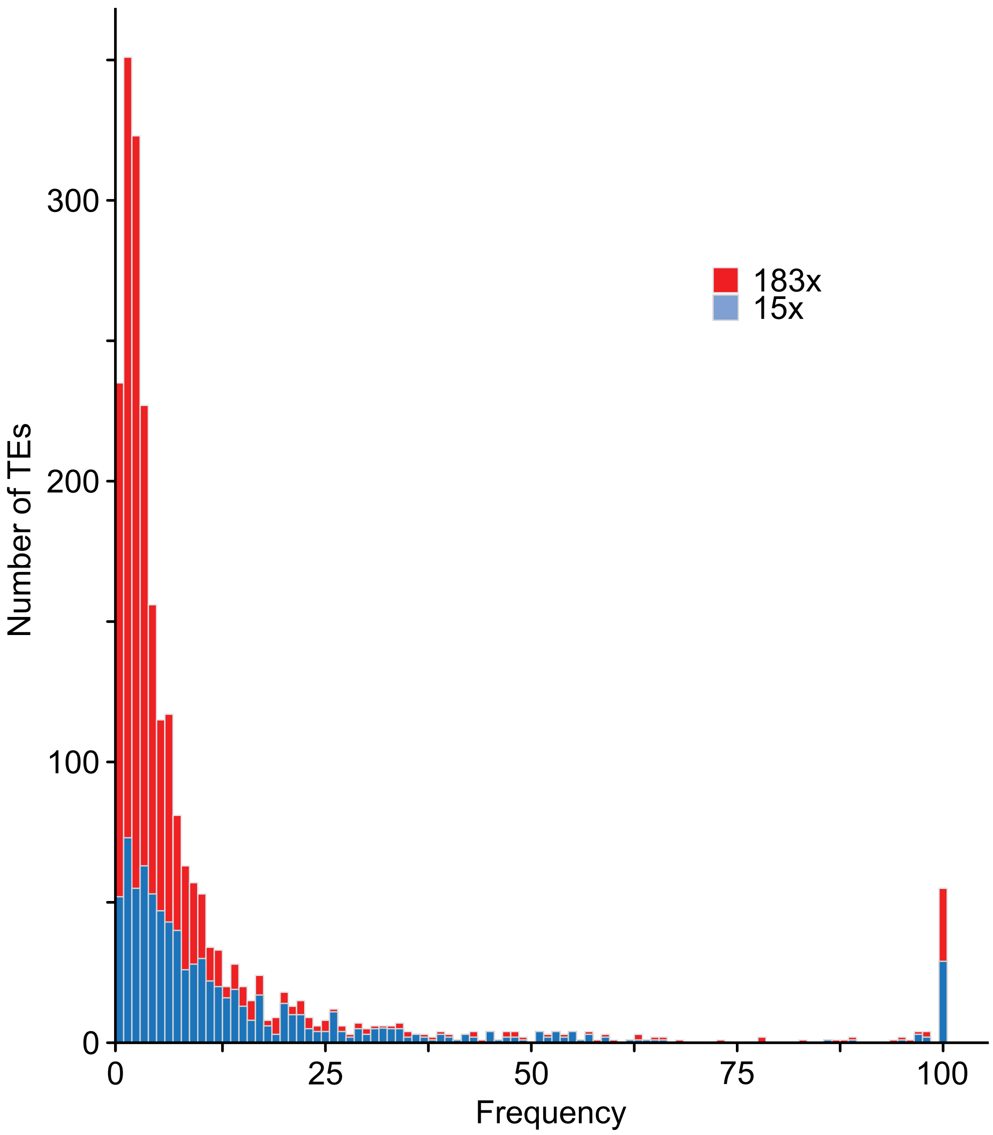

Impact of down-sampling on low frequency TE detection

Frequency distributions of OUTSIDER TE insertions detected in the G73 (183x) dataset (red) and in the 15x dataset (blue).

Table S1 : primer sequences used for TE detection in Figure 3.

**Name of the TE and coodinates in G73 (183x)**	**primer forward**	**primer reverse**	**Size (bp)**
FB-2L_9291436	ATTTTGGCCACTTGTTGGAA	ACGTGGGCAAACGATGTTAT	276
hobo-X_9999330	GCCCCAGAGAAATTCAATCA	TTGTAAGTACCCGCGATGTG	312
hobo-2R_12262307	GACAGGCAAACAAGGAAAGC	AAAAACGGGGTGCCTAGAGT	312
ZAM-2L_16741915	GTCAAGGACTGAAATCCTTTTACT	TTCGCTCAGACAGAACAACG	546
ZAM-2L_16868329	GGAGAGCGGGCACTATTTTA	CCCGGTTCGATGTCTTCTAC	303
ZAM-2R_18388126	CTGCAAAGTTTGTTCGGAGA	TTCGCTCAGACAGAACAACG	466
ZAM-3L_13591033	TTGTGTGTCCTCGTTTCCAG	GCGGTTACGGCATTTTTAAG	937
ZAM-3L_23400613	CATCGTCTAGTACTCCCTGGAAA	TTCGCTCAGACAGAACAACG	539
ZAM-3R_14695230	TTGAATGCTGCCTGAGTGTC	TTCGCTCAGACAGAACAACG	538
gtwin-3L_163676	AGTGGAAACGATCCTCTGGA	CGGGCTCTAAATCAAACTCG	1152
gtwin-3L_6082767	TGGTGTCCTTTGACTTGGTG	ACAGGGCCCCTACTTCAACT	2813
gtwin-3L_19327660	TTGTAAGTACCCGCGATGTG	CGGGCTCTAAATCAAACTCG	1242
412-2R_14285430	AAGATACACGGGCGATGAAG	GCAACAAAGTGCTGCCATAA	213
micropia-3R_1854698	CAAGTTTTCGGCCAATTCAT	ACACCCTGCATTTTCTGAGG	371
blood-2R_10026801	CCAAAATAGACGCATGCAGA	CGTGGGGATGCTGACTTAG	373
blood-X_22439053	AGGGTCCAGAAGAACACCAA	CTCACACCTGTTGTCGCTGT	722
hobo-X_10003439 presence	TTGTAAGTACCCGCGATGTG	ACGGGGAAGTGTAGCGTTTA	384
hobo-X_10003439 excision	CGAGTGTGTGTGTTGGTGTG	ACGGGGAAGTGTAGCGTTTA	343

Table S2 : primer and probe sequences used for digital PCR experiment (resumed in Figure 2).

target	*detection*	*primers*	*seq*
412-2R_14285430	insertion	forward	TTGCTAAACTCCAAATTGCTGG
		reverse	AAGTGCTGCCATAAGTTAATATGC
		probe	56-FAM/TCAATGCGG/ZEN/TGGCCCCAGAAATAA/3IABkFQ
	no insertion	forward	TTGCTAAACTCCAAATTGCTGG
		reverse	ATCATAGAGAAACCAGTGCCAG
		probe	56-FAM/TCAATGCGG/ZEN/TGGCCCCAGAAATAA/3IABkFQ
blood-2L_18128567	insertion	forward	CCCAGAAGTGTTTCGGCAAT
		reverse	CACCCAACTGCAAGGAA
		probe	56-FAM/TATGCCACC/ZEN/AATTGAGAACACGT/3IABkFQ
	no insertion	forward	CCCAGAAGTGTTTCGGCAAT
		reverse	TGGCACGTTACACCTCAA
		probe	56-FAM/ACTCGTCCA/ZEN/CTCAGTGCACGTGCCA/3IABkFQ
roo-2L_13882749	insertion	forward	AAAACCGGAAAGCAACAGG
		reverse	CGGAGCCCAAAATTGTAAGTC
		probe	56-FAM/AGCAGTGAC/ZEN/ACCTTGACTTACATTTTG/3IABkFQ
	no insertion	forward	ACAGGCAAAATGTAAGTCAAGG
		reverse	GCACTAAACATTCGCCATTCAT
		probe	56-FAM/TCGAGTTTT/ZEN/GAATTCATTCCAAGCAG/3IABkFQ
gtwin-3L_163676	insertion	forward	TATTTCTAGTTCCCGCAAGCC
		reverse	CTGTTGCTGACCGTTCGT
		probe	56-FAM/ACGCGCCCA/ZEN/AACTGAGTTCAGCGC/3IABkFQ
	no insertion	forward	AGTGGAAACGATCCTCTGGA
		reverse	GGTGTCTTCAGCAACGTAGT
		probe	56-FAM/TGCCCACGA/ZEN/GCTTGGTCTCTGCCA/3IABkFQ
gtwin-3L_6082767	insertion	forward	CACTCGATGTGCGTATGTGT
		reverse	TATTGTCCCTGGTAGCAGCC
		probe	56-FAM/CCGGTGGTC/ZEN/TCCAGGCGGTGGA/3IABkFQ
	no insertion	forward	CTCGATGTGCGTATGTGTTTG
		reverse	TTATCGCAGACTTTCCCACC
		probe	56-FAM/CATTTTGTT/ZEN/TGCGGCTTTTTTGCG/3IABkFQ/
ZAM-2L_16741915	insertion	forward	AGAGGATTTCTCCAACCCCT
		reverse	GCAGCAAACACTTGTAGACG
		probe	56-FAM/CCTCCGGGG/ZEN/AGTCTTGCGGAGGT/3IABkFQ
	no insertion	forward	AAGAGGAAGAGCGAGAGGAT
		reverse	CCGAGAAAGCCTCGAAAGAA
		probe	56-FAM/TGGCGCGCG/ZEN/CAAGCGTAAGC/3IABkFQ
ZAM-2L_16868329	insertion	forward	CAGATGCATGTGCTCGATTG
		reverse	GTGGTGTATGGTACCGATGG

		probe	56-FAM/CGGCTTAAG/ZEN/CGGAGCCACTCCAATCCC/3IABkFQ
	no insertion	forward	idem IN
		reverse	GTATTCACAGCGTCGTTTCG
		probe	56-FAM/AGGCAGAGC/ZEN/GCGCCCACCCAT/3IABkFQ
ZAM-2R_18388126	insertion	forward	ATTCATCCATCCAGTCAGCC
		reverse	AAATTCTCCCAAGACGACCG
		probe	56-FAM/TGCACGCCG/ZEN/GGGCAAACTGG/3IABkFQ
	no insertion	forward	CAGTGTACGTACCCCTTTCC
		reverse	AAGTGAACCACACACACTCG
		probe	56-FAM/TGCACGCCG/ZEN/GGGCAAACTGG/3IABkFQ
ZAM-3L_13591033	insertion	forward	CTGTTGCTATGCATGTTGCG
		reverse	CCCGGTTCGATGTCTTCTAC
		probe	56-FAM/CCCCTCCCT/ZEN/CTAAGCCACCACGCC/3IABkFQ
	no insertion	forward	GCTTTTGCGCTTCGTCAATC
		reverse	GGCCCGAACACTTGTTAATG
		probe	56-FAM/CGCGCGCAT/ZEN/GCATTAAAGCTGCC/3IABkFQ
RpL32_gene	gene	forward	CACCAGTCGGATCGATATGC
		reverse	CATTTGTGCTGCAAGGAGAC
		probe	5HEX/TGGCACAAT/ZEN/CCTCGTTGGCACTCACCG/3IABkFQ

Table S3 : Impact of sequencing depth on the detection of OUTSIDER TE insertions

	**G73**	**G73 subsampling**					
Depth	183x	145x	115x	92x	76x	38x	15x
**Depth per haplotype**	**0.91x**	**0.72x**	**0.57x**	**0.46x**	**0.38x**	**0.19x**	**0.07x**
OUTSIDER TEs detected<br><br>(% of the original set)	2334<br><br>100%	2295<br><br>98%	2199<br><br>94%	2106<br><br>90%	2026<br><br>87%	1599<br><br>68%	1064<br><br>45%
