# Supplementary material for: TrEMOLO: accurate transposable element allele frequency estimation using long-read sequencing data combining assembly and mapping-based approaches
Source: Genome Biol. 2023 Apr 3;24:63. doi: 10.1186/s13059-023-02911-2 (PMC10069131; doi:10.1186/s13059-023-02911-2)
Supplement: Supplementary file 2 — Additional file 2. Review history. [file 13059_2023_2911_MOESM2_ESM.docx]

Review History

**First round of review**

**Reviewer 1**

**Were you able to assess all statistics in the manuscript, including the appropriateness of statistical tests used?**

There are no statistics in the manuscript.

**Were you able to directly test the software?**

No

**Comments to author:**

Review for "TrEMOLO: Accurate transposable element allele frequency estimation using long-read sequencing data combining assembly and mapping-based approaches"

In this study, the authors developed a tool called "Transposable Element MOnitoring with LOng-reads (TrEMOLO)" which may detect Transposable Element (TE) insertions and deletions between a reference genome and assembly. More important, TrEMOLO may detect the TE insertions in the unmapped/unassembled reads and calculate the allele frequency. The second feature is important for tracking the activity of a transposon or transposons in a population or in somatic tissues. This tool should be useful for people studying active TEs or TE polymorphism in a population. However, I do have concerns about the performance of the tool. The performance does not seem to be great with the simulation data. In the real experimental data, it is difficult to have a comprehensive evaluation.

Major comments

1. The false negative rate is not ideal

Line 199 - 200 "sensitivity was higher for TrEMOLO and TrEMOLO_NA (84.6% and 83.3%, respectively) 931 and 917 true-positive results compared with 813 (73.9%) and 12 (1%) for TLDR and TELR, respectively.".

This means 15% to 17% of the insertions are not detected, which is not ignorable. Moreover, this is simulation data, not real data. Usually tools perform much better on simulation data than on real data (with more complications).

In addition, according to Table 2, TrEMOLO failed to detect a TE insertion with 9.6% allele frequency. In fact, three out of the 8 polymorphic loci confirmed by ddPCR were not detected by TrEMOLO. Certainly I understand the sample number is very small, but this seems to be consistent with the notion that the false negative rate is higher than ideal and there is room for improvement. This would be quite problematic for population studies. As far as I know, the allele frequency of the majority of polymorphic TEs in a population is usually very low. Many of them are only limited to one accession. If someone pooled the DNA for sequencing, I guess TrEMOLO would miss most of those low-frequency insertions.

In the simulated sequence, all the positions of insertions should be available. So I strongly suggest the authors pick up a few false negative loci, and study the intermediate files to see if you could figure out why the tool failed to detect them. It would be great to improve the sensitivity. From the perspective of sensitivity, the advance of this tool seems to be incremental.

2. Lack of data about the detection of deletions or excisions

At the beginning of the result section, the authors mentioned (line 120-122) "the INSIDER TE detection module identifies TE insertions and deletions present in the newly assembled genomes compared with the reference by aligning the two genomes and parsing the pairwise alignment…"

Line 172 - 173 "TrEMOLO also lists the putative INSIDER TE deletion events,…"

Polymorphic TEs could be due to recent transposition events as well as deletions of existing TEs. (See Transposable elements contribute to dynamic genome content in maize

Sarah N. Anderson,Michelle C. Stitzer,Alex B. Brohammer,Peng Zhou,Jaclyn M. Noshay,Christine H. O'Connor,Cory D. Hirsch,Jeffrey Ross-Ibarra,Candice N. Hirsch,Nathan M. Springer)

Besides insertions, excisions also reflect the activity of a transposon, so it is important to detect both insertions and deletions including excisions. However, in the subsequent result sections, the authors never talked about the performance of TrEMOLO with deletions and it does not seem this is designed in the simulation experiment (only insertions are simulated). I assume there are deletions in the real dataset. It would be good to demonstrate this function in the results.

Minor comments

1. It took me some time to realize that an existing TE library is required for TrEMOLO to conduct the analysis (correct me if I am wrong), so it would be good to describe that at the beginning of the results.

A related question is whether the screening of TSD is based on the family of relevant TEs and how you handle the elements with variable lengths of TSD. Please clarify.

2. Line 182 "i.e. clipped reads or partially mapped reads or both"

Here you seem to imply that clipped reads are different from partially mapped reads. Please specify what clipped reads mean.

3. Table 1, it is difficult for me to understand the data with Wtdbg2 + Ragoo. Only less than half of the outside TEs are detected. More than 500 outside TEs are missed. You would assume many of the reads representing minor alleles (with TE insertions) may have been incorporated into the assembly. However, you only detected 39 more outside TEs compared to the better assemblies. The number does not seem to add up. It would be good to discuss why this is the case.

4. Line 464: not sure what "BET" is, please spell it out.

**Reviewer 2**

**Were you able to assess all statistics in the manuscript, including the appropriateness of statistical tests used?**

There are no statistics in the manuscript.

**Were you able to directly test the software?**

No

**Comments to author:**

Mourdas et al describe a novel tool for finding TE insertions based on long-read data. Long-reads hold great promise for TE identification, hence efficient tools that facilitate this task are urgently needed. I'm however not entirely convinced if Tremolo will be my first choice for this task (see comments below). However I commend the authors for providing the tool as SnakeMake pipeline and with a Singularity container. I'm getting more and more convinced that automatic workflow tools (eg Nextflow, Snakemake) combined with containerization (eg Docker, Singularity) will be very promising for bioinformatics, as it could finally help to overcome some of the pressing problems (eg reproducibility crisis).

major comments:

- my main critizism is that the pipeline seems unnecessary complex, which may severely restricting its user-base. Especially the need for an assembly based on the long-reads seems unnecessary to me. Tremolo requires as input a reference assembly (makes perfect sense), long-reads (fine) and an assembly based on the reads (why?). In my lab we have many long-read data and would be very happy to find a tool that enables us to find TE insertions in these data, but the need for an assembly based on the reads lowers my enthusiasm for tremolo. I do not even understand why this is necessary and the authors provide no justification for this step. In the OUTSIDER module, tremolo uses Sniffles to find SVs and then checks if the SVs are TEs, which makes perfect sense to me. But why is this only done for "minor" haplotypes? This would also work for major haplotypes (eg fixed TE insertions) based on the reference assembly and thus making it unnecessary to use the INSIDER module.

Basically, I'm wondering why can I not just use the OUTSIDER module with the reference assembly instead of the long-read assembly. Why is the INSIDER module even necessary? There must be some justification for this, but I can not think of any and the authors provide no reasons either. I'm just a big fan of simple pipelines, simplificity enhances usability, simplifies troubleshooting (eg bugs), throughput/performance, increases reproducibility and repeatability, enhances intuitive understanding of the results and eventually, it will also increase the number of users of a pipeline. This unnecessary complexity now even leads to two artificial categories in their analysis (eg table 1: insider TEs and outsider TEs) that have no biological representation.

- another issue is the validation, especially of the population frequency estimates with Tremolo. They used 20kb reads for validation. However, ONT and PacBio produce very variable read-length (in our hands, unfortunatelly, frequently <7kb) therefore it would be interesting to validate Tremolo esp the population-frequency estimates i) also with shorter reads and ii) mixes of different read-length (again with ONT we receive a wild mix of very diverse read-lengths). With another tool, I evaluated the effect of different read-length and found the read-length had a major impact on the frequency estimates, thats why I think this will be an important validation.

- the authors do not mention one major advantage of long-reads for TE finding. Long-reads enable to differentiate between different variants of a TE: eg many TE families have internal deletions (hobo, P-element), 3'-truncations (non-LTRs) or solo-LTRs. Does the tool provide information on which variation was found (eg internally deleted or full-length)?

I guess not but this is a pity. An additional, but related question, how does the tool handle a case when different variations are segregating at the same insertion sites. For example consider a hobo insertion at chromosome X and position 1211. At this site, 50% of the individuals have the full-length hobo insertion and 50% a major internal-deletion (eg most of the bases). What will the tool report? A fixed full-length hobo insertion?

- 186 - 192: they perform a validation of Tremolo using simulated reads. But they skip one important step of their pipeline, i.e. the assembly of the long-reads. Is it still a valid validation and fair comparision to other tools if an important step of the pipeline is skipped?

- I was wondering about the coordinates of the TEs. The outsider module is finding the TEs with respect to the long-read assembly. Are these coordinates than translated to the reference genome using lift-over (supplementary fig S1 suggests that lift-over is used)?

minor:

- Table 1: for wtdbg2+Ragoo. why is the sum of INSIDER + OUTSIDER not the total number of TEs? Is there some redundancy, eg are there some TEs detected by both INSIDER and OUTSIDER?

- 60: 35% of Dmel are TEs? Not sure how reliable this estimate is, most papers argue it is around 12%.

- Table 1: it is surprising that the largest genome has the smallest BUSCO. BUSCO gives the fraction of completly assembled conserved genes. One would expect that the largest genome has the most complete genes.

- 101 I would write: "may mask some minor ..variations" we had assemblies where to our surprise the minor (not the major) variant was included in the assembly

- 162-164: please rewrite, this sentence is difficult to understand, why are TSDs filtered out and when are they filtered out?

- in my experience, abbreviations reduce readability. maybe just write short-reads instead of SRs

- 310 maybe: its still a challenge due to the repetitive nature of the TEs

- 325: "...without a priory". a priory what? ..without a priory assumptions?

- 352 for the Bergman TEs, I think there is a citation Quesneville 2005 PLoS comp biol

- 374 options -F 2048 and -L, please mention in brackets what they are doing

- 379-382 modifying CIGARs and additing 'N' nucleotides to reads. Why is this necessary and what is this achieving?

**Reviewer 3**

**Were you able to assess all statistics in the manuscript, including the appropriateness of statistical tests used?**

Yes, and I have assessed the statistics in my report.

**Were you able to directly test the software?**

Yes

**Comments to author:**

In " TrEMOLO: Accurate transposable element allele frequency estimation using long-read sequencing data combining assembly and mapping-based approaches," Mourdas et al. present a novel pipeline to annotate transposable elements in newly assembled genomes using long-read sequencing data.

The pipeline consists of three modules. First, long-read-based assemblies are compared to a given reference to identify structural variations (SV) that are subsequently matched against a TE database. The second module is dedicated to identifying TEs that have been left out of the assembly by mapping the long reads and analyzing all that remained unmapped. Finally, an analysis module searches for target site duplications and calculates allele frequencies for each TE insertion. In addition to simulated benchmarks, the authors provide experimental validations of their computational method using a D. Melanogaster Piwi knockdown line published elsewhere.

The presented tool is an exciting contribution and could be valuable to the community. The pipeline design appears to be straightforward and well-motivated. Unfortunately, the paper is a bit hard to read. Also, the article could be improved by adding some additional information.

Major:

- Motivation, introduction, and general exposition of the tool are straightforward. However, the description of the results and the intuition for the individual analysis and benchmark steps could be described and displayed a bit better (see comments on tables, text, and figures below).

- In my view, one of the most critical aspects is the missing exposition of parameters used for the various tools in the TrEMOLO pipeline. The yaml files shipped with TrEMOLO allow the user to adjust some of them. It would be interesting to know which influence this parameterization has. I understand that a complete exploration of the parameter space is impossible. However, I'd like to encourage the authors to discuss some of them in the context of the tool's performance.

- I believe it would be helpful to get some additional information on the runtime and the resources needed by TrEMOLO in real-world scenarios. I'd like to suggest that the authors consider publishing their benchmarking data directly. However, I understand that this is not always possible because of size problems and institutional limitations.

- The coverages used for the experimental validation of the method are quite substantial. I appreciate that ONT coverages of >= 80x-700x may become financially feasible some time for organisms with substantially larger genomes than D. melanogaster. Current genome assemblies, however, frequently rely on much smaller (ONT/PacBio) coverages. While it is clear that the frequency estimation feature (especially for entire populations) depends on a high coverage, the analysis of individual genomes may not require this depth. To demonstrate the applicability of TrEMOLO in those cases, I'd like to suggest simulation scenarios with lower coverages (~20-30x)

Minor:

- Table 2: it is unclear what the second number in some of the fields in the third column means, and I suggest updating the table description accordingly.

- Table 3: I would like to suggest providing other benchmarks in this table (alternatively as a graph). For instance, it would be interesting to know whether the number of outsider TEs not detected in the original set changes as the original data is gradually downsampled. This might be a proxy for the estimation of false positives.

- p. 5, l. 121: genomescompared -> genomes compared

- p. 6, l.140f: In the INSIDER TE detection module, the authors state that "non-reference TE insertions that are found frequently enough among the reads incorporated" thresholds?

- p. 8, l.197fff: The description of the tools' sensitivities and specificities is complicated to read. I suggest showing the relevant measures of sensitivity, specificity, positive predictive value, etc., in Figure 2. The absolute numbers of TEs displayed on a log scale make it difficult to appreciate the differences between TLDR and the TrEMOLO flavors.

- p. 9, l. 203: Please revise the following sentence "The difference between TrEMOLO and TrEMOLO_NA was relevant, and it might be more important if a different reference sequence than the tested sample were used."

- I had difficulties with the singularity installation as the system needed an update of certificates. I was able to fix them by hacking the TrEMOLO/Singularity file:

\# apt dependencies

apt update

apt install -y ca-certificates

apt install -y ...

The authors may like to update their code if this is a general problem.

-Is the Software likely to be of broad utility? Is it easy to install and use?

Yes. Smaller installation problems are addressed in the text above.

-Does the software represent a significant advance over previously published tools (as demonstrated by direct comparison with available related software)?

Based on the provided benchmarks, TrEMOLO represents an advantage over existing software. To better assess the significance of the advantages, some additional analyses are suggested.

-Please indicate briefly the novel features and/or advantages of the software and/or please reference the relevant publications and which alternatives, if any, it should be compared with.

In comparison to existing software such as TLDR and TELR, TrEMOLO shows improvements in terms of false positive and false negative rates. Some of the results have been experimentally validated. Furthermore, it distinguishes itself because it does require ab inito TE detection. However, I would suggest to extent the assessment of the method. Specifically, I would be interested in the tools' performance when using smaller coverages - especially when comparing genome assemblies of individuals (smaller coverage) to their respective reference genomes (or other assemblies).

-Are the methods appropriate to the aims of the study, are they well described, and are necessary controls included?

While the methods and results sections could be improved (see above), the manuscript contains all relevant information to appreciate the method.

-Are the conclusions adequately supported by the data shown?

Yes.

-Are sufficient details provided to allow replication and comparison with related analyses that may have been performed?

See suggestions above.

-Is the paper of broad interest to others in the field, or of outstanding interest to a broad audience of biologists?

As TEs are barely understood, in part, because of the lack of computational methods, the general topic of this paper is of interest to a larger audience.

**Authors Response**

**Point-by-point responses to the reviewers’ comments:**

Comment

*Response*

Reviewer #1: Review for "TrEMOLO: Accurate transposable element allele frequency estimation using long-read sequencing data combining assembly and mapping-based approaches"

In this study, the authors developed a tool called "Transposable Element MOnitoring with LOng-reads (TrEMOLO)" which may detect Transposable Element (TE) insertions and deletions between a reference genome and assembly. More important, TrEMOLO may detect the TE insertions in the unmapped/unassembled reads and calculate the allele frequency. The second feature is important for tracking the activity of a transposon or transposons in a population or in somatic tissues. This tool should be useful for people studying active TEs or TE polymorphism in a population. However, I do have concerns about the performance of the tool. The performance does not seem to be great with the simulation data. In the real experimental data, it is difficult to have a comprehensive evaluation.

*In the new version of the manuscript, and especially with the improvements made to the TrEMOLO pipeline, we hope to have answered most of the criticisms and allayed the reviewer's fears concerning the performance of TrEMOLO.*

Major comments

1.	The false negative rate is not ideal

Line 199 - 200 "sensitivity was higher for TrEMOLO and TrEMOLO_NA (84.6% and 83.3%, respectively) 931 and 917 true-positive results compared with 813 (73.9%) and 12 (1%) for TLDR and TELR, respectively.".

This means 15% to 17% of the insertions are not detected, which is not ignorable. Moreover, this is simulation data, not real data. Usually tools perform much better on simulation data than on real data (with more complications).

*Response: As mentioned by the reviewer, indeed TrEMOLO, at the time of our first submission, did not catch all insertions included in the simulated datasets. As the reviewer, we were concerned by the % of undetected insertions in our simulated dataset. Thus, we decided to improve our TrEMOLO pipeline by giving more weight to the insertions detected by CIGAR and by taking into account the clipped reads. These are reads that are aligned on a large part of their sequence, but their remaining part is not, and the unaligned part of the read is said clipped (see the SAM format specifications).*

*Moreover, in the improved TrEMOLO version, the last version of minimap2 is used (v.2.24) (Line 381 and 451). This version has been described to efficiently align the long INDELs insertion/deletion (see* [*https://doi.org/10.1093/bioinformatics/btab705).*](https://doi.org/10.1093/bioinformatics/btab705).)

*Thanks to these improvements, TrEMOLO and TrEMOLO_NA detect by now 1070 and 1064 true-positive insertions respectively and the false negative rates drop to 3%. Note that no bioinformatic tool has reached such a sensitivity in simulated dataset, as shown, for instance, in our comparison with TLDR and TELR. The manuscript has been updated accordingly (Lines 200 to 208 and Figure 2).*

In addition, according to Table 2, TrEMOLO failed to detect a TE insertion with 9.6% allele frequency. In fact, three out of the 8 polymorphic loci confirmed by ddPCR were not detected by TrEMOLO. Certainly I understand the sample number is very small, but this seems to be consistent with the notion that the false negative rate is higher than ideal and there is room for improvement. This would be quite problematic for population studies. As far as I know, the allele frequency of the majority of polymorphic TEs in a population is usually very low. Many of them are only limited to one accession. If someone pooled the DNA for sequencing, I guess TrEMOLO would miss most of those low-frequency insertions.

*Response: As underlined by the reviewer, in the first version of the manuscript TrEMOLO failed to detect a TE insertion with 9.6% allele frequency. Thanks to the improvement of TrEMOLO explained previously, we are now able to estimate the frequency of this insertion at 17.3%. The Table 2 has been modified in this sense (Line 280 to 288).*

In the simulated sequence, all the positions of insertions should be available. So I strongly suggest the authors pick up a few false negative loci, and study the intermediate files to see if you could figure out why the tool failed to detect them. It would be great to improve the sensitivity. From the perspective of sensitivity, the advance of this tool seems to be incremental.

*Response: As previously mentioned, the new TrEMOLO version improved the sensitivity of the pipeline by almost an order of magnitude (less than 3% of false negatives). As suggested by the reviewer, we checked the 3% remaining missing data reads. We observed that some of these undetected insertions are either inserted in other TE copies or are only supported by clipped reads. The clipped reads are located in complex regions, generally hard to sequence (highly compacted heterochromatin e.g.) or hard to map (highly repeated regions, low complexity ones, incomplete reference sequence) making difficult to detect TEs.*

*Moreover the missing TEs could be explained by the simulation per se. Indeed, it is also important to know that the simulation of the reads can have stochastic biases related to the non-equiprobability of the distribution of the reads obtained on each region of the genome. Indeed, for an average depth of 5x, there may be variability between 10X and 0 for a given region.Thus, in being almost equivalent to a true sequencer, DeepSimulator will also provoke these variations, and some insertions being not covered, they cannot be detected.*

2.	Lack of data about the detection of deletions or excisions

At the beginning of the result section, the authors mentioned (line 120-122) "the INSIDER TE detection module identifies TE insertions and deletions present in the newly assembled genomes compared with the reference by aligning the two genomes and parsing the pairwise alignment..."

Line 172 - 173 "TrEMOLO also lists the putative INSIDER TE deletion events,..."

Polymorphic TEs could be due to recent transposition events as well as deletions of existing TEs. (See Transposable elements contribute to dynamic genome content in maize Sarah N. Anderson,Michelle C. Stitzer,Alex B. Brohammer,Peng Zhou,Jaclyn M. Noshay,Christine H. O'Connor,Cory D. Hirsch,Jeffrey Ross-Ibarra,Candice N. Hirsch,Nathan M. Springer)

Besides insertions, excisions also reflect the activity of a transposon, so it is important to detect both insertions and deletions including excisions. However, in the subsequent result sections, the authors never talked about the performance of TrEMOLO with deletions and it does not

seem this is designed in the simulation experiment (only insertions are simulated). I assume there are deletions in the real dataset. It would be good to demonstrate this function in the results.

*Response: Since we have been working with retrotransposons, we have not focused the manuscript on TE deletions. However, TrEMOLO can indeed detect deletions, when either INSIDERs or OUTSIDERs, but not both at the same time (we will deal with such matter in the future versions). Thus the full mobilization of DNA transposons (excision and insertion) can also be studied using for instance the TrEMOLO INSIDER module. As an example, the revised Table 1 now displays the numbers of predicted TE deletions as suggested by the reviewer. Moreover, in Fig. 3, we used PCR to validate the TrEMOLO prediction of an excision of the hobo DNA transposon in the G73 fly population. The text was modified accordingly (Line 226 to 228).*

Minor comments

1. It took me some time to realize that an existing TE library is required for TrEMOLO to conduct the analysis (correct me if I am wrong), so it would be good to describe that at the beginning of the results.

*Response: We have corrected this point in the revised version of the manuscript according to the reviewer comment and the term “TE library” has now been clearly mention (Line : 110)*

A related question is whether the screening of TSD is based on the family of relevant TEs and how you handle the elements with variable lengths of TSD. Please clarify.

*Response: By default the pipeline relies neither on the identity of the TE nor on the length of the TSD. Moreover, we have added an option “FILE_SIZE_TE_TSD” in order to specify the maximum length of a TSD if it is known.*

2. Line 182 "i.e. clipped reads or partially mapped reads or both"

Here you seem to imply that clipped reads are different from partially mapped reads. Please

specify what clipped reads mean.

*Response: There are two types of reads that do not map perfectly on a genome: the*

*“clipped” reads that do not map at their extremity and the “partially mapped” that do not map internally.*

3. Table 1, it is difficult for me to understand the data with Wtdbg2 + Ragoo. Only less than half of the outside TEs are detected. More than 500 outside TEs are missed. You would assume many of the reads representing minor alleles (with TE insertions) may have been incorporated into the assembly. However, you only detected 39 more outside TEs compared to the better assemblies. The number does not seem to add up. It would be good to discuss why this is the case.

*Response: We are sorry for these misincorporated values in the first version of Table 1. It has been corrected in the revised version of the manuscript.*

4.	Line 464: not sure what "BET" is, please spell it out.
*Response: We have corrected : “EtBr” (line 493).*

Reviewer #2: Mourdas et al describe a novel tool for finding TE insertions based on long-read data. Long-reads hold great promise for TE identification, hence efficient tools that facilitate this task are urgently needed. I'm however not entirely convinced if Tremolo will be my first choice for this task (see comments below). However I commend the authors for providing the tool as SnakeMake pipeline and with a Singularity container. I'm getting more and more convinced that automatic workflow tools (eg Nextflow, Snakemake) combined with containerization (eg Docker, Singularity) will be very promising for bioinformatics, as it could finally help to overcome some of the pressing problems (eg reproducibility crisis).

*Response: We are pleased to note that the reviewer appreciated our usage of automatic workflow tools.*

major comments:

- my main critizism is that the pipeline seems unnecessary complex, which may severely restricting its user-base. Especially the need for an assembly based on the long-reads seems unnecessary to me. Tremolo requires as input a reference assembly (makes perfect sense), long-reads (fine) and an assembly based on the reads (why?). In my lab we have many long-read data and would be very happy to find a tool that enables us to find TE insertions in these data, but the need for an assembly based on the reads lowers my enthusiasm for tremolo. I do not even understand why this is necessary and the authors provide no justification for this step. In the OUTSIDER module, tremolo uses Sniffles to find SVs and then checks if the SVs are TEs, which makes perfect sense to me. But why is this only done for "minor" haplotypes? This would also work for major haplotypes (eg fixed TE insertions) based on the reference assembly and thus making it unnecessary to use the INSIDER module.

Basically, I'm wondering why can I not just use the OUTSIDER module with the reference assembly instead of the long-read assembly. Why is the INSIDER module even necessary? There must be some justification for this, but I can not think of any and the authors provide no reasons either. I'm just a big fan of simple pipelines, simplificity enhances usability, simplifies troubleshooting (eg bugs), throughput/performance, increases reproducibility and repeatability, enhances intuitive understanding of the results and eventually, it will also increase the number of users of a pipeline. This unnecessary complexity now even leads to two artificial categories in their analysis (eg table 1: insider TEs and outsider TEs) that have no biological representation.

*Response: We understand the concerns of the reviewer on these aspects. However, the pipeline itself is really easy to launch, as it is a single command-line with a simple parameter file for end-users. The internal behavior of the pipeline is invisible to end-users, and even if it seems complex, it is not more complex than tools such as RepeatModeler or RepeatMasker internally.*

*A positive impact of adding a complete assembly instead of only relying on mapping has been already reported (Comparison and benchmark of long-read based structural variant detection strategies, Jiadong Lin, Peng Jia, Songbo Wang, Kai Ye, bioRxiv 2022.08.09.503274; doi:* [*https://doi.org/10.1101/2022.08.09.503274*](https://doi.org/10.1101/2022.08.09.503274) *), especially for insertions of long size. Both our*

*simulated and real data could confirm such a gain. They also showed that, using a closest reference (i.e. our own assembly), we increase the mapping rate and thus reduce the false negative discovery rate: switching from the standard dm6 reference genome to our own assembly reduced the unmapped rate from 13.3% to 9.6%.*

*The difficulty of the assembly step has been recently quite reduced by numerous helpful tools,*

*such	as	our	own	CuleBRONT,	(Orjuela	et	al,	2022)*
[*https://github.com/SouthGreenPlatform/culebront)*](https://github.com/SouthGreenPlatform/culebront) *and having a good assembly with long reads for genome of reasonable size is quite easy for a non-highly repeated or very polyploid genome (e.g. we performed chromosome scale assembly of rice genomes for a platinum stage level in less than 5 days in our lab). Finally, having long reads higher than a mean of 25-30kb is also a state-of-the-art in many labs by now (DOI: 10.1111/1755-0998.13308; DOI*[*:10.1016/j.csbj.2021.07.006;*](https://doi.org/10.1016/j.csbj.2021.07.006) [*DOI :10.1016/j.aaf.2021.06.001)*](https://doi.org/10.1016/j.aaf.2021.06.001)*. Even when TrEMOLO cannot leverage the assembly step (when it is not possible to assemble highly complex or polyploid genomes or, in organisms like fungi which contain polysaccharides impairing DNA extraction), and has therefore to rely only on the mapping-based strategy, it still performs better than state-of-the-art tools, as shown by the outputs of TrEMOLO-NA with our simulated data (Figure 2).*

- another issue is the validation, especially of the population frequency estimates with Tremolo. They used 20kb reads for validation. However, ONT and PacBio produce very variable read-length (in our hands, unfortunatelly, frequently <7kb) therefore it would be interesting to validate Tremolo esp the population-frequency estimates i) also with shorter reads and ii) mixes of different read-length (again with ONT we receive a wild mix of very diverse read-lengths). With another tool, I evaluated the effect of different read-length and found the read-length had a major impact on the frequency estimates, thats why I think this will be an important validation.

*Response: As shown below, the mean of 20 kb corresponds to sizes ranging between 870 and 109 000 base pairs in our simulated and experimental data sets. We also recovered the size of reads identifying the TEs, and found that only long reads (i.e. longer than 3.5 kb) were able to detect TEs. This is probably because, with our standard parameters, to support an insertion the reads have to be longer than 80% of the TE size. We understand the reviewer's question, but we note that the current ONT sequencing data usually display an average read length higher than 20kb. As we mentioned earlier, the true key point is in the DNA extraction, in order to obtain long reads. In our datasets, N50 sizes are of 19.65 and 36.06 kb, for instance, and the mean size is**around 15 and 25 kb, respectively.*

*Here are the length distributions of reads from the different simulated datasets we used. As you can see, some reads are quite short while some are long, representing a quite real ONT sequencing.*

*Here is the length distribution of all reads (in green) and of reads that allowed the detection of OUTSIDERS. The latter are longer than the former, probably because the required length for detecting a TE must have a similar size to that of this TE.*

- the authors do not mention one major advantage of long-reads for TE finding. Long-reads enable to differentiate between different variants of a TE: eg many TE families have internal deletions (hobo, P-element), 3'-truncations (non-LTRs) or solo-LTRs. Does the tool provide information on which variation was found (eg internally deleted or full-length)? I guess not but this is a pity.

*Response:TrEMOLO has been designed with the possibility to detect recent events of transposition, and thus full length TEs. While all structural variations are recovered, sequences that do not respect our criteria (80% of total length and 80% of identity in standard options) are filtered out. However, it could be possible to detect such variant structures, by modifying either the parameters or the TE library input file (by adding a dedicated sequence of interest), but we do not guarantee the success of the analysis.*

An additional, but related question, how does the tool handle a case when different

variations are segregating at the same insertion sites. For example consider a hobo insertion at chromosome X and position 1211. At this site, 50% of the individuals have the full-length hobo insertion and 50% a major internal-deletion (eg most of the bases). What will the tool report? A fixed full-length hobo insertion?

*Response: We understand the point raised by the reviewer. If the TE library input file contains only a complete hobo sequence, only almost complete hobo insertions will be identified using the default TrEMOLO parameter (80% of both sequence homology and length). Thus, the 50% with internal deletions will be discarded. However, we tested the detection of internally deleted TEs by adding shorter sequences to the TE library input file, and in this case we were able to recover some.*

- 186 - 192: they perform a validation of Tremolo using simulated reads. But they skip one important step of their pipeline, i.e. the assembly of the long-reads. Is it still a valid validation and fair comparison to other tools if an important step of the pipeline is skipped?

*Response: Benchmarking requires that the parameters are set to the same values for all tools being compared. For instance, since TLDR parameters are fixed, we had to use these to perform a fair benchmarking of all three tools with respect to the mapping efficiency. We also mapped all of the 3 on the same genome sequence, in order to avoid any reference bias and to compare them, everything being equal. In addition, assembly is not performed at all by TrEMOLO, and thus errors in assembly will not be TrEMOLO-related but assembler-related. That’s why we also performed a test of assemblers later in the manuscript.*

- I was wondering about the coordinates of the TEs. The outsider module is finding the TEs with respect to the long-read assembly. Are these coordinates than translated to the reference genome using lift-over (supplementary fig S1 suggests that lift-over is used)?

*Response: Yes, the OUTSIDER TE coordinates determined on the genome assembly are then transposed on the reference genome using* lift-over and the file name is “POS_TE_OUTSIDER_ON_REF.bed”. *We have now modified the figure S1 to clarify this point.*

minor:

- Table 1: for wtdbg2+Ragoo. why is the sum of INSIDER + OUTSIDER not the total number of TEs? Is there some redundancy, eg are there some TEs detected by both INSIDER and OUTSIDER?

*Response: We thank the reviewer for pointing out this error. It has been corrected.*

- 60: 35% of Dmel are TEs? Not sure how reliable this estimate is, most papers argue it is around 12*Response: We thank the reviewer for pointing out this mistake. It has been corrected by around 15% (Line: 51). Note that a significant variation exists regarding these estimates. These differences are likely to be at least partly explained by the genome assembly, or the part of the genome assembly that is analyzed, or both.*

- Table 1: it is surprising that the largest genome has the smallest BUSCO. BUSCO gives the fraction of completely assembled conserved genes. One would expect that the largest genome has the most complete genes.

*Response: BUSCO provides only the level of correctly assembled conserved genomes, but not the level of correct global assembly of the genome itself. The genic fraction that BUSCO estimates is the “easiest” to assemble, while assembling the repeated fraction is more complex, and even can be either incomplete or chimerical. Thus the genic region may be almost completely and correctly assembled, but not the non-genic region. In our dataset, we estimate that N50 is not a good tracker for the best assembly (see for reference* [*https://doi.org/10.1111/1755-0998.13364)*](https://doi.org/10.1111/1755-0998.13364))*. In addition, when comparing the assemblies, we noticed that the Wtdbg2 one has a lower GC-content than the dm6 reference and that the Flye-based assemblies are better in this regard as shown by the BUSCO score, explaining why we did not choose the longest N50 as the metric for our assembly choice. Indeed a choice of good metrics for assembly is quite a big problem, as highlighted in* [*https://link.springer.com/article/10.1186/s12859-020-3382-4.*](https://link.springer.com/article/10.1186/s12859-020-3382-4.)

- 101 I would write: "may mask some minor ..variations" we had assemblies where to our surprise the minor (not the major) variant was included in the assembly

*Response: We corrected according to the reviewer's comment (Line 92).*

- 162-164: please rewrite, this sentence is difficult to understand, why are TSDs filtered out and when are they filtered out?

*Response: We have corrected the text : (Line 156-162)*

*“*If the putative TSDs are located more than 2bp away from the detected 5’ and 3’ ends of the TE insertions, they are filtered out because it could be a simple duplication present in the genome. In addition, for OUTSIDER TE insertions, if the putative TSD sequence is not found at the putative insertion locus as a unique sequence in the assembled genome, it is also filtered out and not considered as the sequence of the empty site . Finally, retained TSDs allow to better define the 5’ and 3’ end of the inserted TE.”

- in my experience, abbreviations reduce readability. maybe just write short-reads instead of SRs

*Response: We have taken into account the reviewer's comment and the revised version has been modified accordingly.*

- 310 maybe: its still a challenge due to the repetitive nature of the TEs *Response: The revised version has been modified (Line 321).*

- 325: "...without a priory". a priory what? ..without a priory assumptions?

*Response: In the revised version, it has been replaced by “* without *ab initio* TE detection*” (Line 337).*

- 352 for the Bergman TEs, I think there is a citation Quesneville 2005 PLoS comp biol

*Response: We use the drosophila-transposons curated database available at* [*https://github.com/bergmanlab/drosophila-transposons.*](https://github.com/bergmanlab/drosophila-transposons) *There is no citation outside of the one related to this repository in our knowledge (Ashburner M., Bayraktaroglu L., Benos T., Chillemi J., Crosby L., de Grey A., Han S., Kaminker J., Lewis S., Liao G., Reese M., Bergman C. (2021). Drosophila transposon canonical sequences (version 10.2). URL:* [*https://github.com/bergmanlab/drosophila-transposons/)*](https://github.com/bergmanlab/drosophila-transposons/))

- 374 options -F 2048 and -L, please mention in brackets what they are doing

*Response: We have mentioned in brackets the definition accordingly to the reviewer’s comment: (Line 393).*

*“*removing any reads with these FLAGS value (see SAM specifications) and overlapping a provided BED file, respectively”

- 379-382 modifying CIGARs and additing 'N' nucleotides to reads. Why is this necessary and what is this achieving?

*Response : To correctly compute frequencies, we need to parse the SAM. For that, we first clean the file using samtools to exclude really problematic reads with FLAGS such as 256 (see sam specs for more details* [*https://samtools.github.io/hts-specs/SAMv1.pdf)*](https://samtools.github.io/hts-specs/SAMv1.pdf) *to remove non-primary alignments. At the same time, we modify the CIGAR to change clipped informations to insertions, as it will provide better support for the insertion vs total depth support. If the CIGAR is still incoherent compared to the read size (eg for hard clipped reads, see sam specs), we also add N at the corresponding location. The text has been modified* (line 392 to 397).

Reviewer #3:

In " TrEMOLO: Accurate transposable element allele frequency estimation using long-read sequencing data combining assembly and mapping-based approaches," Mourdas et al. present a novel pipeline to annotate transposable elements in newly assembled genomes using long-read sequencing data.

The pipeline consists of three modules. First, long-read-based assemblies are compared to a given reference to identify structural variations (SV) that are subsequently matched against a TE database. The second module is dedicated to identifying TEs that have been left out of the assembly by mapping the long reads and analyzing all that remained unmapped. Finally, an analysis module searches for target site duplications and calculates allele frequencies for each TE insertion. In addition to simulated benchmarks, the authors provide experimental validations of their computational method using a D. Melanogaster Piwi knockdown line published elsewhere.

The presented tool is an exciting contribution and could be valuable to the community. The pipeline design appears to be straightforward and well-motivated. Unfortunately, the paper is a bit hard to read. Also, the article could be improved by adding some additional information.

Major:

- Motivation, introduction, and general exposition of the tool are straightforward. However, the description of the results and the intuition for the individual analysis and benchmark steps could be described and displayed a bit better (see comments on tables, text, and figures below).

*Response: We strongly modified the text for results, according to the different reviewers comments.*

- In my view, one of the most critical aspects is the missing exposition of parameters used for the various tools in the TrEMOLO pipeline. The yaml files shipped with TrEMOLO allow the user to adjust some of them. It would be interesting to know which influence this parameterization has. I understand that a complete exploration of the parameter space is impossible. However, I'd like to encourage the authors to discuss some of them in the context of the tool's performance.

*Response: As suggested by the reviewer, we now discuss these parameters in the Discussion (Line 346-356)*

*“In terms of parameters, TrEMOLO in its standard approach will use the previously published 80/80/80 rule for TE recovery (1). Indeed, it relies on 80% of similarity to and 80% in length of the reference TE sequence. However, as shown in the tests before, we could decrease the length coverage down to 10% and still recover true positive variations. This length parameter can thus be modified for e.g. identifying partially transposed elements (for insertions of 5’-truncated LINEs), or insertions of abnormal copies (internally deleted TEs still able to transpose). In the same way, decreasing the level of similarity would allow to detect more distantly related copies from the consensus/reference copy in the database. However, in this case, the decrease of the stringency of detection will have a stronger effect on the quality of results (improving the false positive rates) (45), and we do not recommend it. The best approach would be to add in the reference TE file the targeted sequences instead.”*

*We decided to use the 80/80/80 rule from Wicker et al, 2007, in order to be coherent with the bibliography and the other analyses relying on these parameters. Note that the main restrictive parameter is the percentage of identity, as it will strongly modify the results. Indeed, as shown in Goeta et al, 2003, the similarity level has a much stronger effect on the identification of sequence than the length, especially when the length of the target sequence increases.*

- I believe it would be helpful to get some additional information on the runtime and the resources needed by TrEMOLO in real-world scenarios.

*Response: We have added these informations in the Discussion, (Line 357-360) : “However, as an example, the analyses presented here for simulated data were run on a single laptop computer with 16 Gb of RAM and an 12-cores i7 Intel processor, in less than 10 hours, “*

I'd like to suggest that the authors consider publishing their benchmarking data directly. However, I understand that this is not always possible because of size problems and institutional limitations.

*Response: As suggested by the reviewer, the benchmarking data are now directly available*

*at* [*https://dataverse.ird.fr/dataverse/tremolo_data.*](https://To) *To facilitate the access we performed*

*different links for:*

*the simulated genome* [*https://doi.org/10.23708/DSDTZ0*](https://doi.org/10.23708/DSDTZ0)

*the TrEMOLO 2.2-beta 1 version and the codes used in the paper:*

[*https://doi.org/10.23708/2FYBUL*](https://doi.org/10.23708/2FYBUL)

*the simulated reads : <https://doi.org/10.23708/N447VS>*

- The coverages used for the experimental validation of the method are quite substantial. I appreciate that ONT coverages of >= 80x-700x may become financially feasible some time for organisms with substantially larger genomes than D. melanogaster. Current genome assemblies, however, frequently rely on much smaller (ONT/PacBio) coverages. While it is clear that the frequency estimation feature (especially for entire populations) depends on a high coverage, the analysis of individual genomes may not require this depth. To demonstrate the applicability of TrEMOLO in those cases, I'd like to suggest simulation scenarios with lower coverages (~20-30x)

*Response: We agree with the reviewer that much lower depths than the 60X tested in the simulated dataset should be fairly sensitive enough to detect the presence of TE insertions in individual genomes. This is especially true for homozygous TE insertions calls that should be supported by every read spanning the insertion site. As for the heterozygous TE insertions, even if half of them will be operationally binned into the OUTSIDER class, they should all be supported by about half of the reads. Thus, we simulated a dataset at ~21x, with 100 TE insertions at ~****100%*** *(****homozygous****), 1000 TE insertions at ~****50%*** *(****heterozygous****), and 100 somatic TE insertions at* ***1x*** *so ~****5% (1/21)*** *depth (****somatic insertions****) . We obtained the following results :*

*Homozygous true (+) = 100/100; observed mean-freq=99.73%*

*Heterozygous true (+) = 991/1000; observed mean-freq=53.57%*

*Somatic true (+) = 88/100; observed mean-freq=6.06%*

*All true (+) = 1179*

*All false (+) = 115*

*All false (-) = 21*

*Global sensitivity : 1179/1200 = 98.3%*

*Global specificity : 1179/1294 = 91.1%*

*Moreover, as shown in Figure 6, we subsampled an experimental dataset down to 15X and found that, even at this low depth, 50% of the low frequency insertions were still detected. Interestingly, inspection of the missing insertions showed that they mostly consist in those with the lowest frequencies. Such results were consistent with what we observed in the 21x simulated genome.*

Minor:

- Table 2: it is unclear what the second number in some of the fields in the third column means, and I suggest updating the table description accordingly.

*Response: We have modified Table 2. The two numbers in the last column were the frequencies calculated without and with taking into account the clipped-reads. In the new version, we only reported the TE frequencies estimated by taking into account the clipped-reads as the last version of TrEMOLO does.*

- Table 3: I would like to suggest providing other benchmarks in this table (alternatively as a graph). For instance, it would be interesting to know whether the number of outsider TEs not detected in the original set changes as the original data is gradually downsampled. This might be a proxy for the estimation of false positives.

*Response: In the revised version of the manuscript, we have added a graph showing the impact of the downsampling on the OUTSIDER TE detected (see Figure 6) and the Table 3 is now the Table S3. As we can see, as the depth decreases, more and more TEs are not detected compared to the original dataset.*

- p. 5, l. 121: genomescompared -> genomes compared
*Response: We corrected the text accordingly.*

- p. 6, l.140f: In the INSIDER TE detection module, the authors state that "non-reference TE insertions that are found frequently enough among the reads incorporated" thresholds?

*Response: These TEs are present with a frequency enoughly important to be incorporated in the assembly. Generally, assemblers will build the major haplotype (or randomly one of the two main ones) at each position. We expect then that these TEs have as a mean frequency a value of 50% or more.*

- p. 8, l.197fff: The description of the tools' sensitivities and specificities is complicated to read. I suggest showing the relevant measures of sensitivity, specificity, positive predictive value, etc., in Figure 2. The absolute numbers of TEs displayed on a log scale make it difficult to appreciate the differences between TLDR and the TrEMOLO flavors.

*Response: We understand the difficulty of reading the data and corrected it according to the reviewer’s comment (see new Figure 2).*

- p. 9, l. 203: Please revise the following sentence "The difference between TrEMOLO and TrEMOLO_NA was relevant, and it might be more important if a different reference sequence than the tested sample were used."

*Response: It has been removed, as the sentence itself did not bring any further information.*

- I had difficulties with the singularity installation as the system needed an update of certificates. I was able to fix them by hacking the TrEMOLO/Singularity file:

\# apt dependencies

apt update

apt install -y ca-certificates

apt install -y ...

The authors may like to update their code if this is a general problem.

***Response: Thanks for the reviewer’s comment, we have corrected our Singularity container*** *on the github.*

-Is the Software likely to be of broad utility? Is it easy to install and use? Yes. Smaller installation problems are addressed in the text above.

-Does the software represent a significant advance over previously published tools (as demonstrated by direct comparison with available related software)?

**Based on the provided benchmarks, TrEMOLO represents an adv”antage over existing** software. To better assess the significance of the advantages, some additional analyses are suggested.

-Please indicate briefly the novel features and/or advantages of the software and/or please reference the relevant publications and which alternatives, if any, it should be compared with.

In comparison to existing software such as TLDR and TELR, TrEMOLO shows improvements in terms of false positive and false negative rates. Some of the results have been experimentally validated. Furthermore, it distinguishes itself because it does require ab initio TE detection. However, I would suggest to extent the assessment of the method. Specifically, I would be interested in the tools' performance when using smaller coverages - especially when comparing genome assemblies of individuals (smaller coverage) to their respective reference genomes (or other assemblies).

*Response: as we stated before, we tested with much lower depth, and even in 20x data (see before) and on other single genomes (under preparation on rice individual genomes).*

-Are the methods appropriate to the aims of the study, are they well described, and are

necessary controls included?

While the methods and results sections could be improved (see above), the manuscript

contains all relevant information to appreciate the method.

-Are the conclusions adequately supported by the data shown?

Yes.

-Are sufficient details provided to allow replication and comparison with related analyses that

may have been performed?

See suggestions above.

-Is the paper of broad interest to others in the field, or of outstanding interest to a broad audience of biologists?

As TEs are barely understood, in part, because of the lack of computational methods, the general topic of this paper is of interest to a larger audience.

**Second round of review**

**Reviewer 1**

Review for "TrEMOLO: Accurate transposable element allele frequency estimation using long-read sequencing data combining assembly and mapping-based approaches"
This is a revised version. Overall my comments are well addressed. I only have some minor comments.

1. Line 157 "…the putative TSDs are located more than 2pb away from the detected 5' and 3' ends of the TE…"
"2pb" should be "2bp".

2. Line 198-201 "Using these simulated datasets, we observed that out of the 1100 simulated insertions, TELR and TLDR only detected 12 (1%) and 928 (84.4%) TE true-positive insertions, respectively. By contrast, TrEMOLO_NA found 1064 (96.7%) TE true-positive insertions, illustrating the greater sensitivity of the TrEMOLO mapping algorithm (Fig. 2)."
I noticed that in the last version of the manuscript the true positive was "813 (73.9%)" for TLDR. So the number here (928, 84.4%) is much better than that from last version.
I understand that the TrEMOLO program is improved from last version to this version. However, it seems very unusual that the results for the control program TLDR is also improved, so please clarify.

**Reviewer 2**

I have a comment to:
Response: As shown below, the mean of 20 kb corresponds to sizes ranging between 870 and 109 000 base pairs in our simulated and experimental data sets. We also recovered the size of reads identifying the TEs, and found that only long reads (i.e. longer than 3.5 kb) were able to detect TEs. This is probably because, with our standard parameters, to support an insertion the reads have to be longer than 80% of the TE size. We understand the reviewer's question, but we note that the current ONT sequencing data usually display an average read length higher than 20kb. As we mentioned earlier, the true key point is in the DNA extraction, in order to obtain long reads. In our datasets, N50 sizes are of 19.65 and 36.06 kb, for instance, and the mean size is around 15 and 25 kb, respectively.

This minimum read length is an important information for potential users.
Please mention this limitation of Tremolo in the manuscript, ie. that the tool solely works for reads longer than 4kb.

**Reviewer 3**

The authors have addressed all my comments and concerns.

**Authors Response**

**Point-by-point responses to the reviewers’ comments:**

Reviewer #1: Review for "TrEMOLO: Accurate transposable element allele frequency estimation using long-read sequencing data combining assembly and mapping-based approaches"

This is a revised version. Overall my comments are well addressed. I only have some minor comments.

1.Line 157 "...the putative TSDs are located more than 2pb away from the detected 5' and 3' ends of the TE..." "2pb" should be "2bp".

*Response: Thanks for the mistake pointing, we corrected it.*

2.Line 198-201 "Using these simulated datasets, we observed that out of the 1100 simulated insertions, TELR and TLDR only detected 12 (1%) and 928 (84.4%) TE true- positive insertions, respectively. By contrast, TrEMOLO_NA found 1064 (96.7%) TE true-positive insertions, illustrating the greater sensitivity of the TrEMOLO mapping algorithm (Fig. 2)." I noticed that in the last version of the manuscript the true positive was "813 (73.9%)" for TLDR. So the number here (928, 84.4%) is much better than that from last version. I understand that the TrEMOLO program is improved from last version to this version. However, it seems very unusual that the results for the control program TLDR is also improved, so please clarify.

*Response: Indeed, we recomputed the different values to be the closest possible to the TrEMOLO 2.2 conditions. The increased value for TLDR seems to be linked to the better mapping of minimap2 in its new version.*

Reviewer #2: I have a comment to:

“Response: As shown below, the mean of 20 kb corresponds to sizes ranging between 870 and 109 000 base pairs in our simulated and experimental data sets. We also recovered the size of reads identifying the TEs, and found that only long reads (i.e. longer than 3.5 kb) were able to detect TEs. This is probably because, with our standard parameters, to support an insertion the reads have to be longer than 80% of the TE size. We understand the reviewer's question, but we note that the current ONT sequencing data usually display an average read length higher than 20kb. As we mentioned earlier, the true key point is in the DNA extraction, in order to obtain long reads. In our datasets, N50 sizes are of 19.65 and 36.06 kb, for instance, and the mean size is around 15 and 25 kb, respectively.

This minimum read length is an important information for potential users. Please mention this limitation of Tremolo in the manuscript, ie. that the tool solely works for reads longer than 4kb.”

*Response: We added such a limitation in the discussion part.*
